# Repeated Impact Response of Normal- and High-Strength Concrete Subjected to Temperatures up to 600 °C

**DOI:** 10.3390/ma15155283

**Published:** 2022-07-30

**Authors:** Sallal R. Abid, Ahmmad A. Abbass, Gunasekaran Murali, Mohammed L. J. Al-Sarray, Islam A. Nader, Sajjad H. Ali

**Affiliations:** 1 Department of Civil Engineering, Wasit University, Kut 52003, Iraq; mohmmad1999500@gmail.com (M.L.J.A.-S.); geerweer1415@gmail.com (I.A.N.); sajhali.wasit@gmail.com (S.H.A.); 2 Building and Construction Materials Department, Southern Technical University-Shatrah Technical Institute, Shatrah 64007, Iraq; ahmed.abdulhadi@stu.edu.iq; 3 Peter the Great St. Petersburg Polytechnic University, 195251 Saint Petersburg, Russia; murali_22984@yahoo.com

**Keywords:** repeated impact, ACI 544-2R, high temperatures, high-strength concrete, impact number

## Abstract

With the aim of investigating the response of concrete to the dual effect of accidental fire high temperatures and possible induced impacts due to falling fragmented or burst parts or objects, an experimental work is conducted in this study to explore the influence of exposure to temperatures of 200, 400 and 600 °C on the responses of concrete specimens subjected to impact loads. Cylindrical specimens are tested using the recommended repeated impact procedure of the ACI 544-2R test. Three concrete mixtures with concrete nominal design strengths of 20, 40 and 80 MPa are introduced to represent different levels of concrete strength. From each concrete mixture, 24 cylinders and 12 cubes are prepared to evaluate the residual impact resistance and compressive strength. Six cylindrical specimens and three cubes from each concrete mixture are heated to each of the three levels of high temperatures, while the other six cylinders and three cubes are tested without heating as reference specimens. The test results show that the behavior of impact resistance is completely different from that of compressive strength after exposure to high temperatures; the cylindrical specimens lose more than 80% of the cracking and failure impact resistance after exposure to 200 °C, while impact resistance almost vanishes after exposure to 400 and 600 °C. Concrete compressive strength is found to be effective on the unheated impact specimens, where the higher-strength cylinders retain significantly higher impact numbers. This effect noticeably decreases after exposure to 200 and 400 °C, and vanishes after exposure to 600 °C.

## 1. Introduction

Concrete is one the construction materials that is known for its adequate safe endurance when subjected to high-temperature exposures. Compared to metallic constructions, concrete structural members can last for a longer time under the catastrophic destruction of accidental fires. However, the degradation of the material’s microstructure is inevitable, which is directly translated into strength losses and unfavorable serviceability issues [1]. Therefore, the assessment of the residual strength and residual structural performance of fire-damaged structural members is a crucial task for civil engineers after each structural fire incident. A trusted post-fire structural evaluation is not an easy task; it requires an adequate knowledge of the multi-scale effects of high temperatures on material and structural states. The structural decision maker needs to be a specialist that depends not only on self-experience, but mainly on the available standards and norms which are generally built based on trusted research works. Therefore, the continuous investigation of fire influence on concrete and reinforced concrete structures is encouraged due to continuous developments in construction materials that impose different performances under high-temperature exposure.

Concrete is significantly affected by high-temperature exposure when the temperature exceeds 400 °C; below this temperature, strength losses are recoverable and damages can be controlled, and the rehabilitation of the structure becomes the favorable choice. When concrete is subjected to temperatures lower than 200 °C, its compressive strength keeps close to the original unheated strength, either slightly higher or slightly lower [1,2]. The strength rise at this range of temperature is attributed to the evaporation of pore water [3,4]. However, a small-to-moderate decrease has been reported in tensile strength and related physical properties where failure is more related to tensile stresses than compressive stresses, such as shear strength or flexural strength [5,6,7,8,9,10,11]. A serious change takes place after exposure to temperatures exceeding 400 °C, which results in microstructural damages that initiate a steep drop in the strength and durability of concrete and concrete structures [12,13]. The expansion of concrete aggregate has a destructive effect on its strength, while the shrinkage of cement paste deteriorates the cement matrix and seriously influences its endurance under loading. The reversed thermal movements of the aggregate particles (expansion) and cement matrix (contraction) have a destructive effect on the chemical and physical bond forces on the mutual surfaces. Hence, the individual strengths of the cement matrix and aggregate and the bond between them decline after exposure to temperatures of 400 °C and higher due to their thermal movements [14,15,16]. Another significantly influencing factor that further accelerates the deterioration of concrete at this temperature is the dehydration of hydrated products [15,16,17]. Most of the available literature reveals significant strength losses after exposure to temperatures higher than 500 °C, at which point the tensile strength, flexural strength and shear strength exhibit effective reductions and the compressive strength significantly drops [3,6,18]. On the other hand, the impact resistance of concrete has been reported to suffer great and quick deterioration even after exposure to temperatures lower than 400 °C, both under low-velocity impact [19,20] and high-strain rate impact [21,22,23,24].

Impact resistance is the ability of the material or the structural member to withstand low- or high-speed impact forces within a very short period of time. The impact resistance of concrete can be evaluated using different testing techniques. One of the most common test methods to evaluate the impact performance of concrete is the drop-weight impact test. In this test, a specific mass is left to drop freely from a specific height and the structural response of the tested element is recorded in terms of impact force, deflection, vibration and possibly strain. Such a type of test is an expensive one and is intended to be performed on moderate- to large-scale test structural elements (beams or slabs), and is a very useful technique to study structural response under the effect of heavy falling objects [25,26,27,28,29]. Another common impact test is the Charpy pendulum test that is used to estimate the effect of impact forces on different sizes of elements that are made of different types of materials [20,21,22,23,24,25,26,27,28,29,30,31,32]. On the other hand, projectile [33,34,35,36,37] and explosive impacts [38,39] are also types of impact forces that are investigated using special, very costly techniques. None of the mentioned tests consider repeated impact influence and none can be termed as simple or low-cost testing methods. Therefore, the ACI 544-2R [40] introduces an impact test methodology that is intended to evaluate the response of concrete materials under repeated low-velocity impacts. For instance, such impact forces are expected in structures such as multi-story parking garages, where a collision impact from the parking vehicles is repeatedly expected on columns and walls.

The ACI 544-2R [40] repeated impact test has been adopted by many research works during the current and last two decades to investigate the capability of different types of concrete to withstand repeated falling-mass impacts. Several previous studies conducted experimental tests using the ACI 544-2R repeated impact procedure to study the response of self-compacting concrete where plain and fiber-reinforced self-compacting concrete mixtures were investigated [41,42,43,44,45,46,47,48,49,50,51]. The concrete grade of the investigated concrete mixtures also varied from normal strength [41,42,43,44,45,46] to high strength [46,47,48,49,50,51]. Rich recent literature is also available on the evaluation of impact performance of preplaced aggregate concrete, with or without fibers, using the ACI 544-2R repeated impact test, where repeated impact tests were conducted on single-layer specimens [52,53,54,55,56], double-layer specimens [56,57,58,59], and triple-layer specimens [56,60]. The incorporation of intermediate glass fiber meshes between the preplaced aggregate concrete layers has also been investigated by some recent research works [58,59,60]. Other researchers used the ACI 544-2R procedure to investigate the repeated impact response of superior concrete such as high-performance and ultra-high-performance concrete [61,62,63] in addition to modern cementitious materials such as engineered cementitious composites [49,64]. On the other hand, some previous studies have investigated the residual repeated impact strength of concrete mixtures incorporating recycled concrete aggregate or other recycled materials and byproducts such as cementitious materials, fillers or fibers [43,46,47,48]. In previous works, experimental ACI 544-2R repeated impact tests have shown that the crumb rubber as a waste material when incorporated into concrete mixtures can improve the impact energy capacity of the concrete mixture [19,44,65].

As introduced in the brief literature survey, the ACI 544-2R has been adopted recently by several research works to cost-effectively explore and compare the impact energy absorption capacity of concrete mixtures. The simple test procedure is used to evaluate the effect of the incorporation of different materials on the response of concrete under impact loads. However, all of the reviewed works considered the normal ambient environment conditions, while very limited research works [19,66,67,68] are available on the repeated impact performance of concrete after fire exposure. A possible loading scenario that may arise during accidental fires is the destruction of some structural and nonstructural parts due to the failure to sustain high temperatures. Consequently, some fragments may fall repeatedly on the lower-level heated structural elements, imposing impact stresses on already fire-deteriorated concrete. Therefore, the evaluation of concrete’s post-fire repeated impact performance is a required study. The most used concrete in building construction is normal-strength concrete. However, the strength of the concrete of old structures might be reduced with time to be lower than usual, while some high-rise buildings are usually constructed using high-strength concrete. There are few previous research studies that have investigated the post-fire residual repeated impact of concrete focusing on the design grade of concrete. Therefore, this study is designed to experimentally highlight the effect of concrete grade on its repeated impact performance at ambient temperatures and after exposure to high temperatures. For this purpose, three concrete mixtures with three levels of design grades were prepared, heated to different high temperature levels, and tested under the ACI 544-2R repeated impact procedure. The concrete mixtures were designed to be of moderately low-strength, normal-strength and high-strength plain concrete.

## 2. Experimental Works

### 2.1. Concrete Mixtures and Materials

The experimental work of this research included preparing three concrete mixtures with three levels of design grades: M20, M40 and M80. These concrete mixtures were designed to achieve 28-day compressive strengths of 20, 40 and 80 MPa, respectively. The per-cubic meter weights of cement, water, fine aggregate and coarse aggregate of the three concrete mixtures are listed in Table 1. A small quantity of super plasticizer was adopted in the concrete mixture M80 to achieve the required acceptable workability. From each of the adopted concrete mixtures, 24 cylindrical specimens of 150 mm diameter and 63 mm thickness were cast to carry out repeated impact tests, while twelve 100 mm cube specimens were cast to conduct the compressive strength tests. The specimens were all water-cured for 28 days using thermally controlled water-curing tanks at a temperature of 20 ± 2 °C.

**Figure 1:**
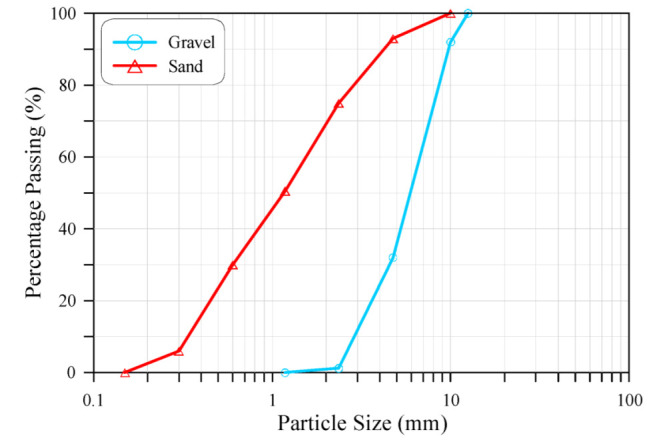
Sieve analysis of the adopted fine and coarse aggregates.

Ordinary Portland cement type 42.5 from Mass Cement Plant, Sulaimaniya, Iraq was adopted in all concrete mixtures with a specific gravity of 3.15 and a specific surface of 368 m^2^/kg. Crushed gravel with a maximum size of aggregate of 12.5 mm with the gradings shown in Figure 1 was incorporated in the concrete mixture as a coarse aggregate, while natural river sand with the grading shown in Figure 1 was used as a fine aggregate. The use of a high quantity of cement and a low water/cement ratio in the M80 concrete mixture imposed the use of the Sika^®^ ViscoCrete 5930-L (Dubai, UAE) super plasticizer to achieve the required mixture workability, while there was no need for this material in the M20 and M40 concrete mixtures.

### 2.2. Heating of Test Specimens

**Figure 2:**
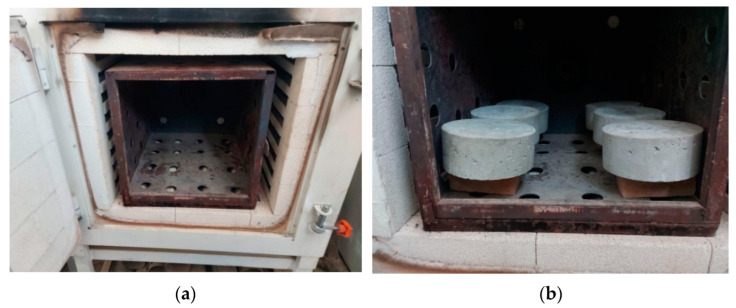
Heating of test specimens: (**a**) the used electrical furnace; (**b**) specimen heating.

**Figure 3:**
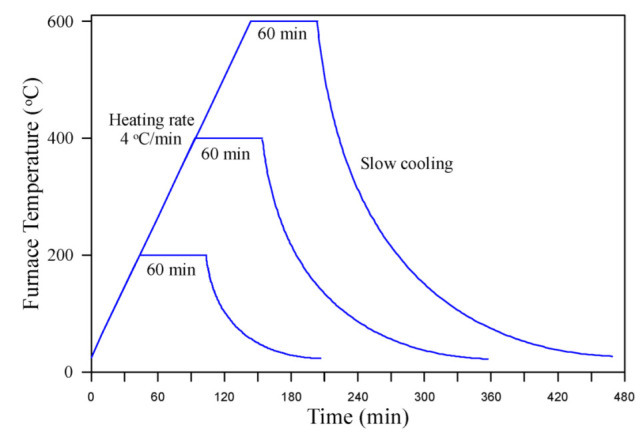
Temperature–time curve of the heating process.

The furnace shown in Figure 2a provided from Ankarin, Ankara, Turkey was facilitated in this study to conduct the heating process of the specimens to the desired high temperatures, as shown in Figure 2b. The furnace was electrically operated and could reach temperatures of approximately 1200 °C. Before heating, the specimens were subjected to a drying process for 24 h using an electrical oven at a temperature of 105 °C. This process was recommended by many previous studies [69,70,71] to avoid specimen spalling inside the furnace owing to the presence of humidity in the specimens. The specimens were heated to three levels of high temperatures, which were 200, 400 and 600 °C. Six cylinders and three cubes from each concrete mixture were heated to each level of elevated temperature, while another group of six cylinders and three cubes were tested without heating as reference unheated specimens. As illustrated in Figure 3, the specimens were initially heated at an approximately constant rate of heating of 4 °C/min [66,67,68,72,73] until reaching the target level of temperature (200, 400 or 600 °C). Then, afterwards, a saturation period of 60 min was adopted to assure that the cores of the heated specimens reached the desired temperature [74,75]. Finally, the door of the furnace was carefully opened so that a phase of natural slow cooling to room temperature was started.

### 2.3. Impact Testing

**Figure 4:**
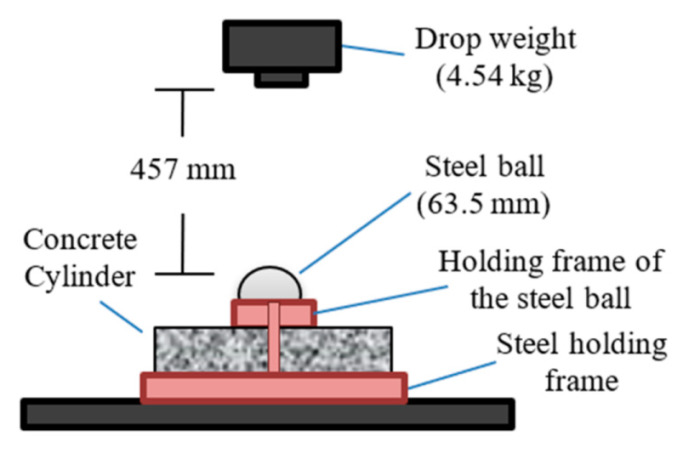
Illustration of the ACI 544-2R repeated impact apparatus.

The repeated impact testing procedure recommended by the American Concrete Institute ACI 544-2R [40] was followed to conduct the impact tests of this study. This procedure is described in the illustration shown in Figure 4. The test was conducted on a concrete cylindrical specimen with a diameter of approximately 150 mm and a thickness of approximately 63 mm. The specimen was held to a stiff steel baseplate using a steel holding frame to prevent its rebound during the impact of a freely dropped mass of 4.54 kg. This drop mass was manually lifted to a fixed drop height of 457 mm and was dropped on a steel ball with a diameter of 63.5 mm resting on the top surface of the test cylinder. The steel ball itself was also held in place using a special steel frame that was connected to the specimen’s holding frame. The test required that the impact was repeated until a surface crack could be visually inspected. At this stage, the number of impacts reached to cause the surface cracking was recorded as the cracking impact number (Ncr). The impact was then continued until the splitting of the specimen, which indicated its failure. The number of impacts at this stage was recorded as the failure impact number (Nf). It should be mentioned that the ACI 544-2R defines failure as the number of impacts required to split the specimens so that they touch at least three of the four vertical holding lugs of the steel holding frame.

**Figure 5:**
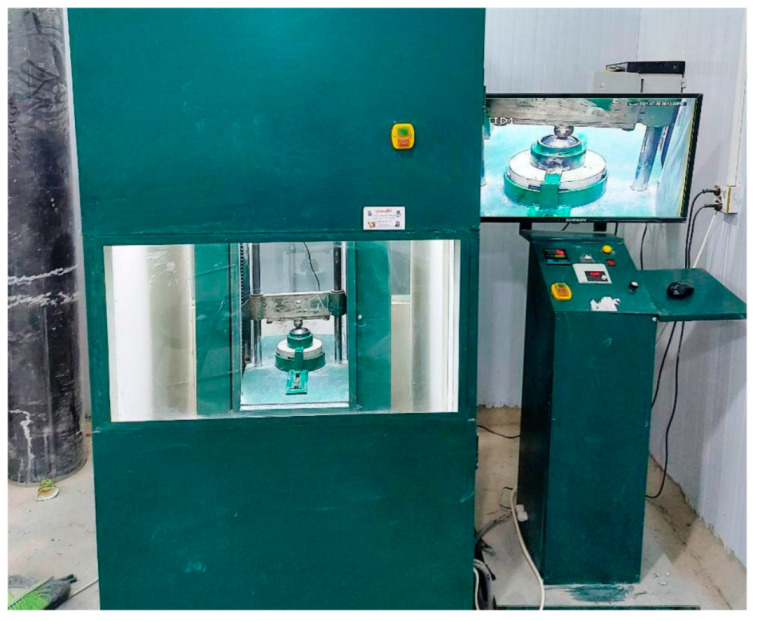
The repeated impact automatic testing machine.

Previous works [61,62,76] have shown that the test requires extensive effort and time to complete the testing of one specimen. Therefore, instead of the manually operated ACI 544-2R test apparatus, an electrical automatic impacting machine was manufactured at Wasit University by the research group for this purpose. The impact testing machine shown in Figure 5 was adopted in this study to conduct the repeated impact tests according to the ACI 544-2R procedure, while a laser counter and an accurate digital camera were used to accurately record the number of impacts and to observe the cracking and failure of the specimens.

## 3. Results and Discussion of Compressive Strength

**Figure 6:**
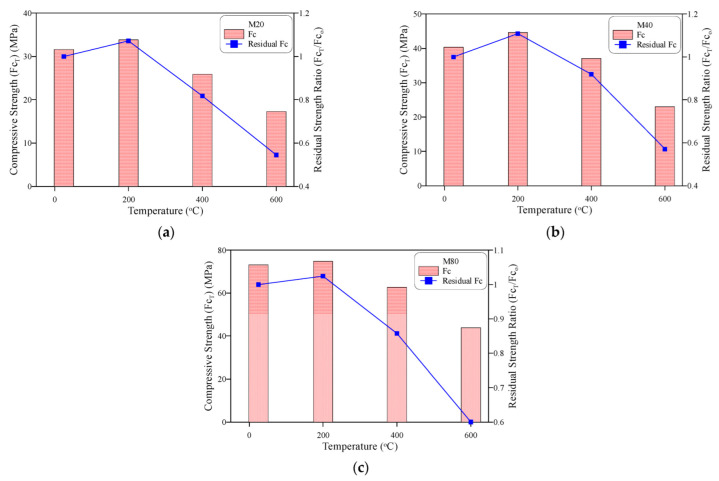
Compressive strength at different temperatures: (**a**) M20, (**b**) M40, (**c**) M80.

The compressive strength test was the only mechanical control test adopted in this study to discuss the quality of the concrete mixtures. As preceded in the previous section, cube specimens were cast, cured and heated under the same conditions of the impact cylindrical specimens. The responses of the compressive strength of each of the three concrete mixtures with low, normal and high design strengths with temperature are shown in Figure 6. It can be seen that the three concrete mixtures exhibited a strength increase after being exposed to 200 °C. Percentage increases compared to the unheated specimens of 2.5 to 9% were recorded for the three concrete mixtures, as shown in Figure 6a–c. Previous researchers attributed the strength increase to the accelerated moisture release from the cement gel surfaces that led to higher Vander Wall’s attraction forces between these surfaces [77]. Abrams [1] reported that the aggregate type had a great impact on the behavior of concrete exposed to high temperature, and that specimens incorporating siliceous aggregate, as adopted in this study, exhibited a strength increase at approximately 300 °C, while such an increase was not recorded for sand–lightweight concretes. Nikolai and Zoldeners [78] attributed the aggregate effect to its occupied size in the concrete mixture. After exposure to 400 °C, the three concrete mixtures exhibited strength reductions that were generally less than 20% of the original strength, while exposing the specimens to 600 °C resulted in a significant decrease in the compressive strength. The residual compressive strength records of the M20, M40 and M80 specimens compared to their corresponding unheated cubes were approximately 55, 57 and 60%. Thus, a significant strength reduction of not less than 40% occurred after exposure to 600 °C. The reduction in strength after 400 °C is attributed to the effective microstructural thermal-induced changes that led to the degradation of cement paste and the interfacial bond with aggregate, and the decomposition of hydrated products initiated at approximately 400 °C by the dehydration of Ca(OH)_2_ into CaO, which was accompanied by the drying and shrinkage of cement gel particles due to the liberation of water, which increased the porosity and deteriorated the strength [79]. Because aggregate has different thermal properties and coefficients of thermal expansion, its thermal movements differ from those of cement matrixes, which leads to a quick bond deterioration after 400 °C, adding another source of strength degradation [6,7,79].

**Figure 7:**
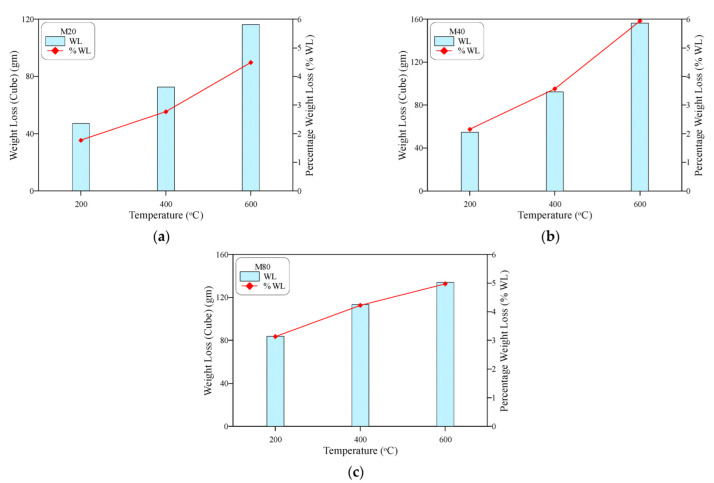
Weight loss of the heated cubes: (**a**) M20, (**b**) M40, (**c**) M80.

The weight loss of the cube specimens shown in Figure 7 assures the obtained results; it is clear in the figures that the three concrete mixtures exhibited an increasing weight loss with the increase in exposed temperature. The percentage weight loss of the three concrete mixtures was in the range of 1.8 to 3.1% at 200 °C, while it increased to a range of 2.8 to 4.2% at 400 °C and a range of 4.5 to 5.9% at 600 °C. The free water in the aggregate–cement matrix voids evaporated around a temperature of approximately 100 °C, while the loss of that absorbed into the fine pores of the aggregate and gel particle structures led to a further weight loss at higher temperatures. Finally, the liberation of the chemically bound water in the hydrated cement products decreased the specimens’ weight after exposure to temperatures between 400 and 600 °C.

## 4. Effect of High Temperatures on the Concrete Impact Specimens

**Figure 8:**
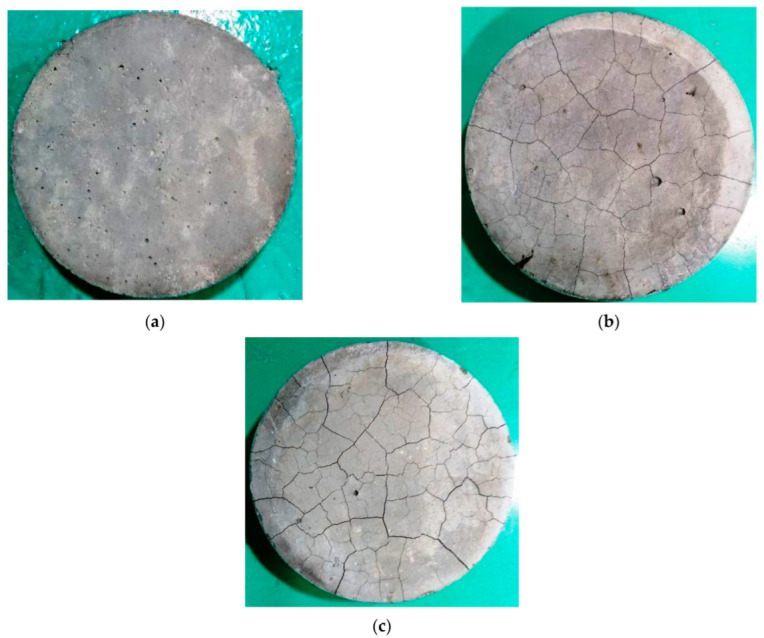
Surface thermal cracking of cylindrical specimens before impact testing: (**a**) 200 °C, (**b**) 400 °C, (**c**) 600 °C.

**Figure 9:**
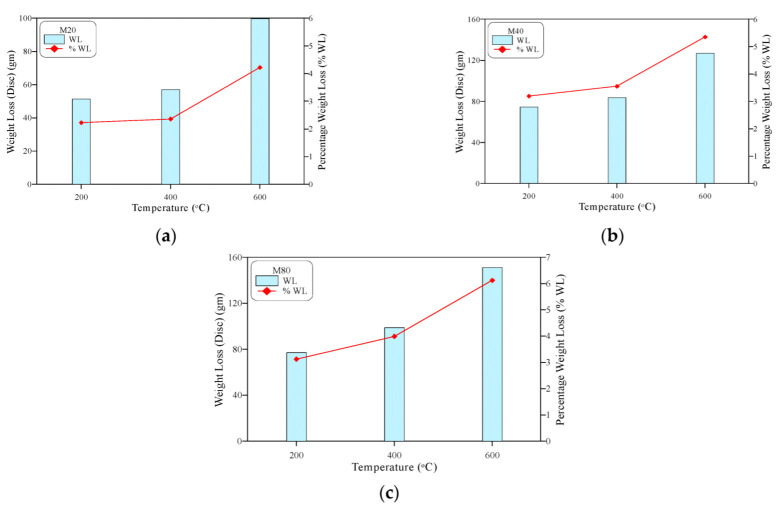
Weight loss of the heated cylinders: (**a**) M20, (**b**) M40, (**c**) M80.

Figure 8 shows pictures for the heated cylindrical specimens before testing. It can be seen clearly in Figure 8a that the exposure to 200 °C did not cause visible thermal deformations on the surfaces of the heated specimens, which is attributed to the limited negative effect of this temperature on the internal microstructure of these samples, as discussed earlier. The loss of the voids’ free water, and even that in the capillary pores, did not result in huge thermal changes in the cement paste or aggregate particles. On the other hand, the further moisture liberation and reversed thermal movements after exposure to 400 °C resulted in the thermal cracking shown in Figure 8b. The dehydration of the hydrated cement products of the specimens heated to 600 °C liberated the chemically bound water, resulting in the significant shrinkage of cement paste which was translated as excessive service cracking, as shown in Figure 8c. The percentage weight losses of the cylindrical specimens shown in Figure 9 confirm the increase in moisture loss as the elevated exposure temperature increased. The figure shows that the percentage weight losses of the cylindrical specimens were in the ranges of 2.23 to 3.19% at 200 °C, 2.36 to 3.99% at 400 °C, and 4.22 to 6.12% at 600 °C.

## 5. Results and Discussion of Repeated Impact Test

### 5.1. Influence of Mixture Strength on the Impact Resistance

**Figure 10:**
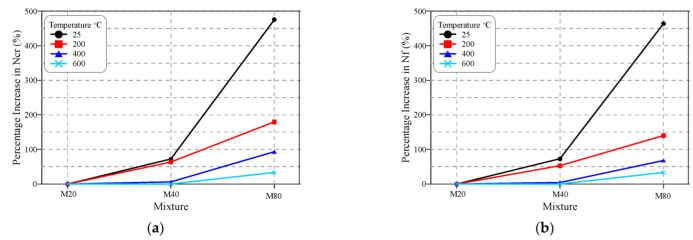
Percentage increase in impact numbers of M40 and M80 compared to M20: (**a**) Ncr, (**b**) Nf.

Figure 10 shows the effect of the concrete mixture grade (compressive strength) on the retained repeated impact numbers. Both Figure 10a,b show that, before heating, increasing the design strength from M20 to M40 resulted in a significant increase in the cracking and failure impact numbers, while the percentage increase for the concrete mixture M80 (with a nominal design strength of 80 MPa) compared to the reference concrete mixture (M20) with the nominal design strength of 20 MPa was significantly higher. For the unheated specimens, the percentage increase in Ncr and Nf of the M40 specimens compared to the corresponding M20 ones ranged from approximately 72 to 73%, respectively. On the other hand, the percentage increase in the high-strength specimens (M80) compared to the corresponding (M20) specimens jumped to the range of approximately 464 to 476%. This was an expected result, as a higher-strength concrete means a stiffer concrete mixture with a higher resistance to the applied compressive impacts and their induced tensile stresses. Thus, with the increase in the mixture’s compressive strength, the specimens can absorb higher impact energies before cracking and failure, which raises the retained numbers of sustained impact blows till cracking and failure. In a previous study on self-compacting concrete [51], it was revealed that, for plain specimens, the retained cracking and failure numbers increased by approximately 29 to 71% for a concrete mixture with 83 MPa compressive strength compared to another with 51 MPa strength. Abid et al. [50] showed that the impact resistance of plain self-compacting specimens under flexural impact increased by approximately 42% as the compressive strength increased from approximately 55 MPa to 83 MPa. On the other hand, the results obtained by an earlier study [8] revealed much higher percentages of cracking and failure impact of 277 and 253% as the compressive strength of the plain specimens increased from 41.3 MPa to 55.6 MPa. The variation in these results highlights the need to investigate the effect of the design strength of plain concrete on its impact resistance, which was one of the main aims of this study.

The effect of the design strength of the impact specimens on their cracking and failure impact resistance decreased with temperature increase, as explicitly revealed in Figure 10. The percentage increase in Ncr of M40 over M20 decreased from approximately 72% at room temperature to 64, 6 and 0% after exposure to 200, 400 and 600 °C, respectively, while the percentage increase in Nf of the same concrete mixture decreased from to approximately 53, 5 and 0%, respectively. On the other hand, exposing the specimens to 200, 400 and 600 °C, decreased the percentage increase in the Ncr of M80 over M20 from approximately 476% to 180, 94 and 33%, respectively, and decreased Nf from 464% to 140, 68 and 33%, respectively. These results are directly related to the discussed graded thermal deteriorations in concrete that increase with the increase in temperature. As concrete deteriorates harder, the differences between the different concrete mixtures becomes narrower as the cement matrix loses most of its original strength that distinguishes it from lower-strength mixtures. Adding this effect to the deterioration of the aggregate particles and the almost complete loss of bonds between the surrounding cement matrix at 600 °C, the number of impact blows required to crack (Ncr) the M20, M40 and M60 specimens were only 1, 1 and 1.3, which were the same numbers of blows required to fail the specimens (Nf). Hence, the specimens of all concrete mixtures heated to 600 °C failed after only approximately one impact blow due to the harsh effect of high temperature exposure, which diminished the differences between the impact resistances of the three concrete mixtures.

### 5.2. Degradation of the Impact Resistance after High-Temperature Exposure

**Figure 11:**
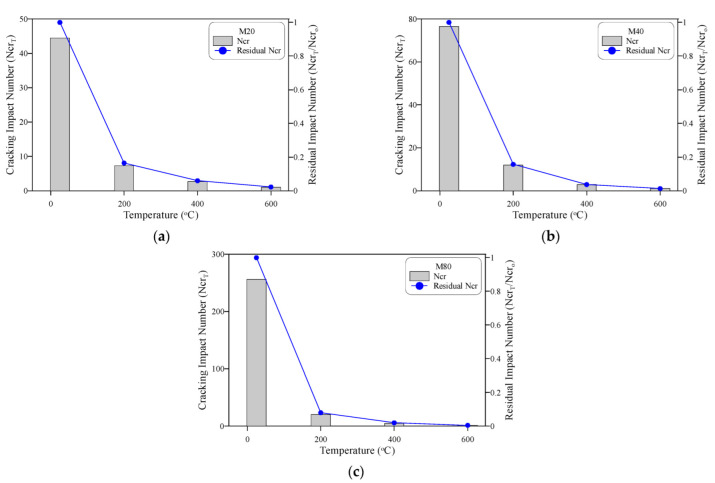
Cracking impact number at different temperatures: (**a**) M20, (**b**) M40, (**c**) M80.

**Figure 12:**
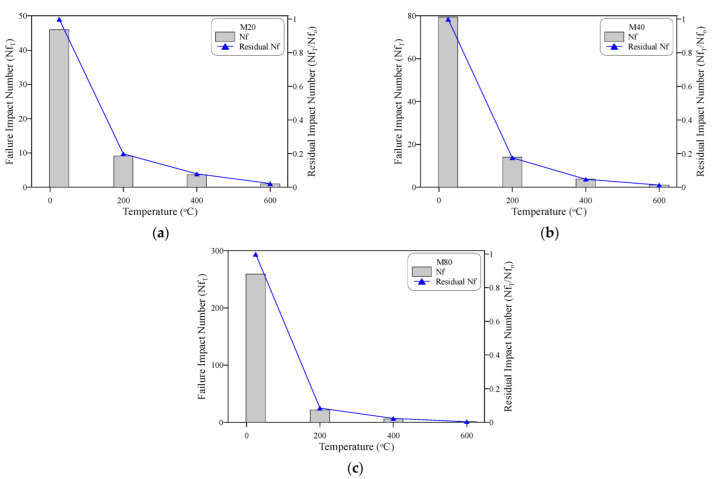
Failure impact number at different temperatures: (**a**) M20, (**b**) M40, (**c**) M80.

The deterioration of the impact resistance of the three concrete mixtures M20, M40 and M80 with temperature is separately depicted for Ncr in Figure 11 and Nf in Figure 12. The huge drop in impact resistance after high-temperature exposure is very obvious in the figures, even at a level of only 200 °C. The Ncr of the M20 specimens shown in Figure 11a decreased from 44.5 blows to as low as 7.3, 2.7, and only 1 blow after exposure to 200, 400 and 600 °C. Thus, the residual records of these concrete mixtures were only 16.5, 6.0 and 2.2% of their original cracking impact resistance before heating. This means that this concrete mixture lost approximately 84% of its impact resistance after exposure to a temperature of 200 °C, while the impact resistance was almost diminished for specimens exposed to 600 °C. Similar results were obtained for the cracking impact resistance of the M40 and M80 mixtures, as shown in Figure 11b,c, where the residual Ncr records of M40 specimens were 15.7, 3.7 and 1.3% after exposure to 200, 400 and 600 °C, respectively. The corresponding residual Ncr records of M80 were even lower at only 8.0, 2.0 and 0.5%, respectively. Similar deterioration sequences were also recorded at the failure stages, as depicted in Figure 12, where the three concrete mixtures retained only 8.4 to 19.9%, 2.4 to 8.0%, and 0.5 to 2.2% after exposure to 200, 400 and 600 °C, respectively.

The excessive deterioration of the repeated impact resistance of the heated specimens is attributed to the induced stresses and stress distribution in the microstructure under the effect of each single impact blow. The compressive impacts concentrated on the center of the specimen’s top surface via the steel ball were diagonally spread across the subsurface layers as multidirectional tensile stress waves that affected the microstructure within a very short period of time. Hence, tensile shocking waves were transferred along all diagonal directions of the specimens. As the heated specimens were already weakened due to the induced thermal stresses that affected the strength of the cement paste, aggregate particles and the bond between them, the action of the shocking tensile waves noticeably accelerated the microstructural degradation and the specimen fracturing. It should be mentioned that the behavior of heated concrete under tensile stresses (splitting tensile test and bending test) is different from that under compressive stresses; several previous studies have reported a faster deterioration of the tensile strength of concrete compared to compressive strength [6,7,14,80]. However, the repeated strength deterioration rates of all control tests are not comparable to that recorded for the repeated impact test, which is, as discussed above, attributed to the different effects of quasi-static and impact loads. Such a quick deterioration of repeated impact records of specimens subjected to high temperatures has also been reported in previous studies for ECC specimens [66,67] and rubberized fibrous specimens [19].

**Figure 13:**
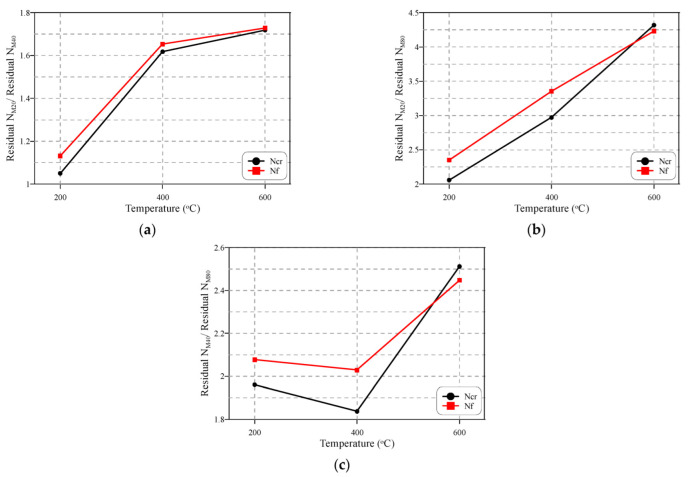
Effect of concrete strength on the degree of deterioration of impact resistance: (**a**) residual M20/M40, (**b**) residual M20/M80, (**c**) residual M40/M80.

It should be noticed that although all concrete mixtures strongly deteriorated after high-temperature exposure, the higher strength concrete exhibited higher percentage losses than the lower-strength concretes. Figure 13 shows the normalized percentage residual Ncr and Nf of the lower-strength concrete mixtures compared to the higher-strength ones. The figure compares the percentage residual impact numbers of the M40 and M80 specimens with those of the M20 ones, as shown in Figure 13a,b, and those of M80 with the residual percentages of M40 shown in Figure 13c. It is obvious that the impact resistance losses of the M40 and M80 specimens were higher than those of the M20 ones. The residual M20/residual M40 ratio was in the range of 1.05 to 1.73 for both Ncr and Nf and increased with the increase in temperature, as shown in Figure 13a. The deterioration of M80 was higher than M40 and was much higher than M20. As depicted in Figure 13b, the percentage residuals of Ncr and Nf of M20 were at least twice those of M80 and reached more than four times those of M80 at 600 °C. Comparing M80 with M40, the percentage residual impact numbers were in the range of 1.84 to 2.51. Hence, it can be said that the percentage residual impact strength of M40 was approximately twice that of M80. The higher reduction in the higher-strength concrete is attributed to its denser microstructure and lower porosity that retard the free fluent of evaporated moisture and induce additional internal vapour pressure that accelerates the deterioration [81,82,83]. The higher quantity of cement is also among the reasons for the higher degradation of M80 over the other concrete mixtures, as the cement matrix occupies a larger volume and thus the dehydration of hydrated cement components induces greater damage in the microstructure.

### 5.3. Failure Patterns of the Impact Cylindrical Specimens

**Figure 14:**
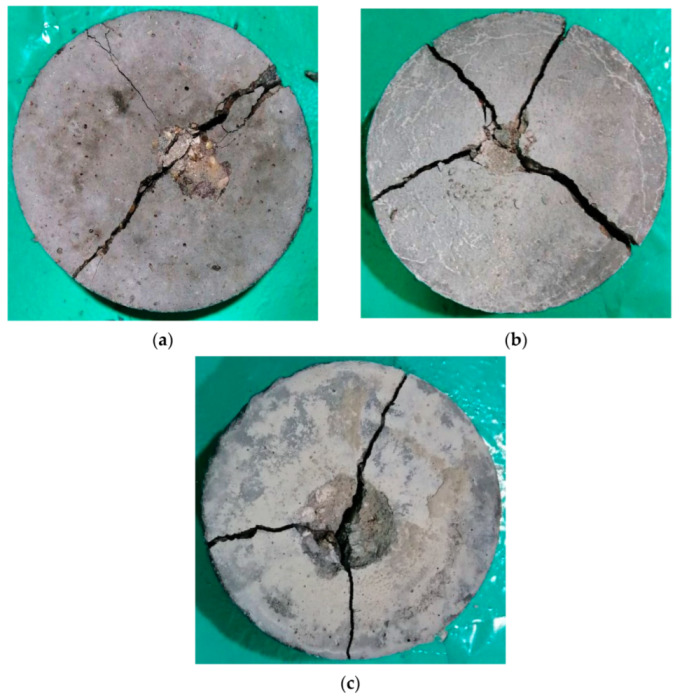
Failure patters of unheated cylindrical specimens: (**a**) M20, (**b**) M40, (**c**) M80.

**Figure 15:**
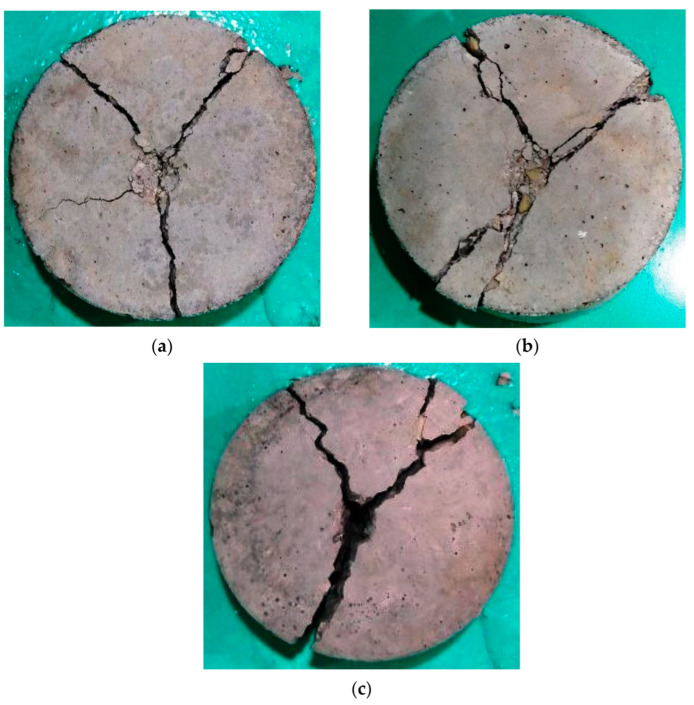
Failure patterns of cylindrical specimens heated to 200 °C: (**a**) M20, (**b**) M40, (**c**) M80.

**Figure 16:**
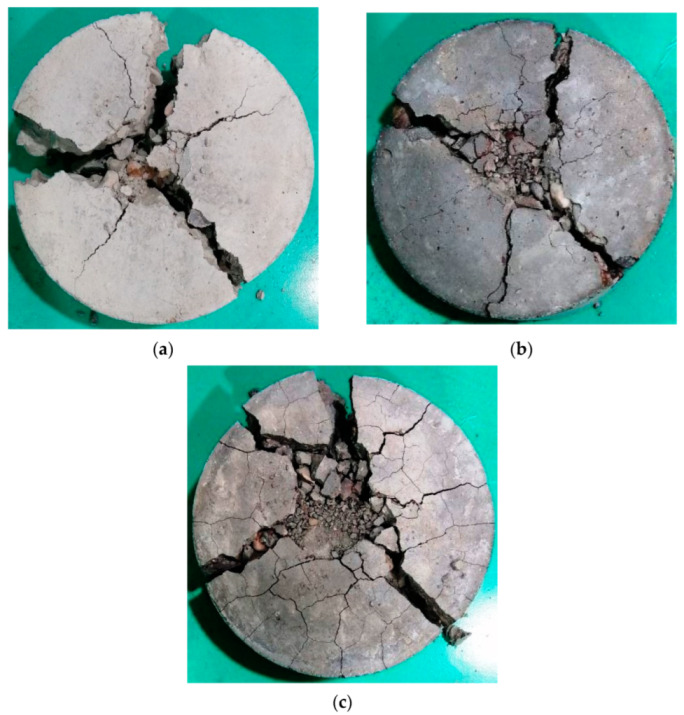
Failure patterns of cylindrical specimens heated to 400 °C: (**a**) M20, (**b**) M40, (**c**) M80.

**Figure 17:**
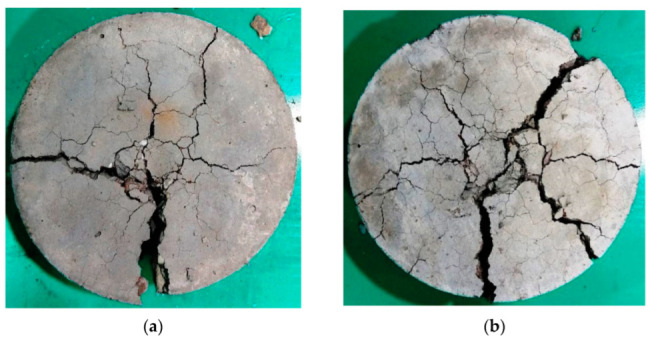

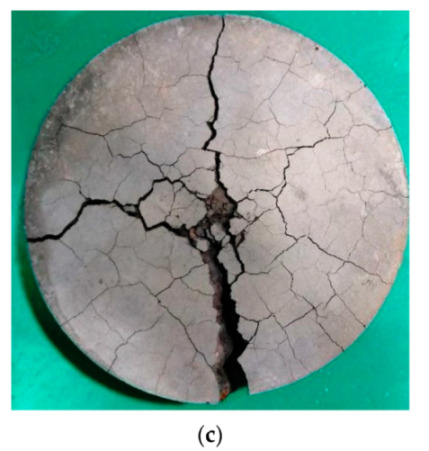
Failure patterns of cylindrical specimens heated to 600 °C: (**a**) M20, (**b**) M40, (**c**) M80.

Samples of the final patterns of the tested cylindrical specimens are shown in Figure 14, Figure 15, Figure 16 and Figure 17 for unheated reference specimens and others tested after exposure to 200, 400 and 600 °C, respectively. The unheated specimens exhibited the usual failure of plain concrete cylinders subjected to repeated impact. As shown in Figure 14, the failure was diagonal, splitting the specimen into two, three or four parts. As the concrete was not reinforced with any kind of reinforcing elements, the cracks were formed after several impact blows along the diameter of the cylinder, as shown in Figure 14a, or diagonally along any possible direction, starting from the central point of impact towards the outer perimeter of the specimen’s top surface, where mostly three or four main diagonal cracks were formed. A few additional impacts were enough to open the previously initiated surface cracks and cause the failure. The received impacts via the steel ball were transferred as tensile radial waves along all directions. As concrete is not a homogenous material, the weaker paths are cracked under the concentration of stresses from the repeated impacts. Cracks then become wider, causing the failure of the specimen with the effect of the continuous impact loads. The weak paths are those that can sustain lower stresses compared to the other parts of the concrete volume which are formed due to weak aggregate particles or a higher porosity and a weaker bond with the cement matrix. Approximately similar failure patterns were also recorded for specimens heated to 200 °C, as shown in Figure 15, but with more minor cracking or fracturing. As discussed in Section 4 and Figure 8, the heating to 200 °C did not cause clear surface thermal deformations, which explains the similar failure patterns to those of unheated specimens.

Oppositely, the specimens heated to higher temperatures were noticeably cracked before testing due to the effect of temperature loss and the other chemical changes occurred within the concrete microstructure after exposure to 400 and 600 °C. Therefore, the surface and the interior structure of the specimens were weakened to a degree that changed their failure patterns to those shown in Figure 16 and Figure 17. The thermal cracks are shown on the surfaces of the specimens exposed to 400 °C, as shown in Figure 16, and are more obvious on those exposed to 600 °C, as shown in Figure 17. As the surface of the specimen was already weakened, the central zone under the steel ball exhibited more pronounced fracturing compared to the unheated specimens. Due to the fractured or destroyed central zone, which is clear in Figure 16b,c, and the already existing thermal cracks, several multidirectional cracks appeared and the specimens failed in less uniform patterns compared to the unheated specimens. The original concrete grade had an effect on the failure before heating, while after heating to high temperatures, all specimens were degraded to a very high degree so that the differences were ineffective. For the unheated tests, the M80 specimens could withstand a significantly higher number of impact blows compared to the lower-strength specimens, which is attributed to the higher stiffness and lower porosity of the microstructure due to the higher cement content. As a result, the central impact zone could sustain a larger fracturing diameter before failure compared to the M20 and M40 specimens, as is clear in Figure 14c.

## 6. Conclusions

With the aim of evaluating the residual repeated impact responses of different grade concrete after exposure to high temperatures, cylinders and cube specimens from three concrete mixtures (M20, M40 and M80) were tested before and after exposure to 200, 400 and 600 °C. From the experimental records obtained in this study and within the limitations of the adopted parameters, the following conclusions can be drawn:

1. Regardless of their original strength, the compressive strength of the concrete cubes exposed to a temperature of 200 °C was in general higher than the original unheated strength, where a strength gain of 2.5 to 9% was recorded for the tested specimens. On the other hand, exposing the cubes to a temperature of 400 °C decreased the strength by no more than 20%, while a strength reduction of not less than 40% was recorded for cube specimens heated to 600 °C;
2. The impact resistances of the impact specimens increased with the increase in the mixture’s compressive strength. Compared to the M20 specimens, the cracking and impact numbers of the M40 specimens (with a nominal design strength of 40 MPa) increased by approximately 72%, while the M80 specimens (with an 80 MPa design strength) retained higher cracking and impact numbers by more than 460%, compared to the M20 specimens;
3. Owing to the degradation of the cement matrix, aggregate particles, and the interfacial bond, the effect of the design compressive strength on the impact resistance of the heated specimens decreased as temperature increased. For instance, the percentage increase in the failure impact resistances of the M80 specimens over the M20 ones decreased from 464% before heating to 140 and 68% after exposure to 200 and 400 °C, respectively. The influence of the original strength faded after exposure to 600 °C, where all concrete mixtures cracked and failed after approximately one impact blow;
4. Owing to the different types of induced stresses, the deterioration in the impact resistance was much higher than that of the compressive strength. The specimens lost more than 80% of their cracking and failure impact resistances after exposure to only 200 °C, while the impact resistance was almost vanished after exposure to 600 °C. For instance, the failure impact numbers of the three concrete mixtures decreased by 80.1 to 91.6%, 92 to 97.6% and 97.8 to 99.5% when exposed to 200, 400 and 600 °C, respectively;
5. Exposing the impact cylindrical specimens to 200 °C did not cause noticeable thermal effects on the surface of the specimens, while a noticeable thermal cracking was visually clear for specimens heated 400 °C, and was more obvious on the surfaces of specimens heated to 600 °C. Therefore, the failure patterns of the cylindrical specimens exposed to 200 °C were similar to those of unheated cylinders with two, three or four diagonal cracks and insignificant central fracturing. This was different for specimens heated to 400 and 600 °C with larger surface fracturing and non-uniform surface cracking;
6. The failure of specimens heated to 400 and 600 °C was not affected by the original concrete strength of the specimens, while this effect was clear for the unheated specimens. As the higher-strength specimens (M80) contained higher amounts of cement, their microstructures were stiffer with lower porosity compared to the lower-strength specimens. As a result, the M80 specimens could withstand much higher impact blows concentrated on the central area of the top surface of the specimens, which caused a larger surface central fracturing zone compared to the M40 and M20 specimens;
7. The available literature on this topic is very limited; therefore, we recommend extending the presented work using different concrete mixtures that include frequently used additives and fibers. For instance, the influence of cementitious materials such as silica fume and fly ash on the impact response of fired specimens could be investigated in future works. Other concrete mixtures with high cementitious material content such as high-performance concrete might behave differently under such kinds of dual thermal and impact loading.

## Figures and Tables

**Table 1:** Materials of the three mixtures (kg/m^3^).

Material (kg/m^3^)	M20	M40	M80
Cement	270	450	709.5
Sand	600	675	648
Gravel	1080	990	870
Water	189	207	189.2
SP	-	-	4.44

## Data Availability

Not applicable.
